# Birth Preparedness and Complication Readiness among Pregnant Women in Southern Ethiopia

**DOI:** 10.1371/journal.pone.0021432

**Published:** 2011-06-22

**Authors:** Mesay Hailu, Abebe Gebremariam, Fissehaye Alemseged, Kebede Deribe

**Affiliations:** 1 Sidam Zone Health Office, Hwassa, Ethiopia; 2 Population and Family Health Department, College of Public Health and Medical Science, Jimma, Ethiopia; 3 Department of Epidemiology and Biostatistics, College of Public Health and Medical Science, Jimma University, Jimma, Ethiopia; 4 College of Public Health and Medical Science, Jimma University, Jimma, Ethiopia; Institute for Clinical Effectiveness and Health Policy (IECS), Argentina

## Abstract

**Background:**

Birth preparedness and complication preparedness (BPACR) is a key component of globally accepted safe motherhood programs, which helps ensure women to reach professional delivery care when labor begins and to reduce delays that occur when mothers in labor experience obstetric complications.

**Objective:**

This study was conducted to assess practice and factors associated with BPACR among pregnant women in Aleta Wondo district in Sidama Zone, South Ethiopia.

**Methods:**

A community based cross sectional study was conducted in 2007, on a sample of 812 pregnant women. Data were collected using pre-tested and structured questionnaire. The collected data were analyzed by SPSS for windows version 12.0.1. The women were asked whether they followed the desired five steps while pregnant: identified a trained birth attendant, identified a health facility, arranged for transport, identified blood donor and saved money for emergency. Taking at least two steps was considered being well-prepared.

**Results:**

Among 743 pregnant women only a quarter (20.5%) of pregnant women identified skilled provider. Only 8.1% identified health facility for delivery and/or for obstetric emergencies. Preparedness for transportation was found to be very low (7.7%). Considerable (34.5%) number of families saved money for incurred costs of delivery and emergency if needed. Only few (2.3%) identified potential blood donor in case of emergency. Majority (87.9%) of the respondents reported that they intended to deliver at home, and only 60(8%) planned to deliver at health facilities. Overall only 17% of pregnant women were well prepared. The adjusted multivariate model showed that significant predictors for being well-prepared were maternal availing of antenatal services (OR = 1.91 95% CI; 1.21–3.01) and being pregnant for the first time (OR = 6.82, 95% CI; 1.27–36.55).

**Conclusion:**

BPACR practice in the study area was found to be low. Effort to increase BPACR should focus on availing antenatal care services.

## Introduction

Childbirth is a universally celebrated event yet for many thousands of women each day, child bearing is experienced not as the joyful event as it should be [1]. Globally 40% or more of pregnant women may experience acute obstetric problems. The WHO estimates that 300 million women in the developing world suffer from short-term or long-term illness brought about by pregnancy and childbirth. Most of maternal deaths occur in the developing world [2]–[6]. For example from 342,900 maternal deaths in 2008, 52% occurred in sub Saharan Africa [7]. This is because of several reasons one of which is inadequacy or lack of birth and emergency preparedness, which is a key component of globally accepted safe motherhood programs. Birth preparedness helps ensure that women can reach professional delivery care when labor begins and reduces the delays that occur when women experience obstetric complications [8]–[11].

Birth Preparedness and Complication Readiness (BPACR) is the process of planning for normal birth and anticipating the actions needed in case of an emergency [12], [13]. Birth preparedness is a strategy to promote the timely use of skilled maternal care, especially during childbirth, based on the theory that preparing for childbirth reduces delays in obtaining this care. A birth plan/emergency preparedness plan includes identification of the following elements: identifying a skilled birth attendant; identifying the location of the closest appropriate care facility; funds for birth-related and emergency expenses; transport to a health facility for the birth and obstetric emergency; and identification of compatible blood donors in case of emergency. The role of BPACR improving the use and effectiveness of key maternal and neonatal services is through reducing delays in deciding to seek care in two ways. First, it motivates people to plan to have a skilled provider at every birth. If women and families make the decision to seek care before the onset of labor, and they successfully follow through with this plan, the woman will reach care before developing any potential complications during childbirth, thus avoiding the first two delays completely. Second, complication readiness raises awareness of danger signs thereby improving problem recognition and reducing the delay in deciding to seek care [14], [15]. Making arrangements for blood donors is also important because women giving birth may need blood transfusions in the event of hemorrhage or cesarean section. Blood donor systems at the community level can help overcome problems related with access to blood [13], [15].

There are evidences from Nepal, Burkina Faso and India [16]–[18] that promoting BPACR improves preventive behaviors, improves knowledge of mothers about danger- signs, and leads to improvement in care-seeking during obstetric emergency. However there are no evidences which clearly indicate the reduction of neither maternal nor neonatal mortality. In Ethiopia, the levels of maternal mortality and morbidity are among the highest in the world and the current estimate of Maternal Mortality Rates is 580 per 100, 000 live births [7] and it is reported that Maternal deaths accounted for 21 percent of all deaths to women age 15–49 [19]. In Millennium Development Goal 5, countries have committed to reducing the maternal mortality ratio by three quarters between 1990 and 2015. Following the commitment with the goal, Ethiopia is expected to reduce maternal mortality in 2015 to 267 maternal deaths per 100,000 live births [20].

Despite the fact that birth preparedness and complication readiness is essential for further improvement of maternal and child health little is known about the current magnitude and influencing factors in Ethiopia. This study therefore aims to fill this gap by assessing the current status and factors associated with birth preparedness and complication readiness among pregnant women in Aleta Wondo Woreda Sidama zone, Ethiopia, through a community based cross sectional study. It is hoped that the results of the study will provide valuable information for design of possible programs and interventions to improve maternal and neonatal health. And also serve as baseline information for further study.

## Materials and Methods

### Study area

Sidama zone is one of the 13 zones found in the Southern Nations, Nationalities and People Regional Government (SNNPRG) with the total population estimate for 2007 was 2,855,386 [21]. Of this 2,610,439 (91%) are rural and 244947 (9%) are urban dwellers [21]. In the zone there are 19 woredas (equals district in other countries) and 2 town administrations. By ethnic group majority are Sidama and the major religious groups are protestant Christians.The potential health service coverage of the zone in 2005 was 38% [22], [23]. During the survey period the number of pregnant women was 119837 (4.26%) and the antenatal care coverage was 69.3%, [23], [24]. According to demographic health survey 2005 only 6% deliveries in Ethiopia occurred in health facilities assisted by skilled health providers [25].

The study was conducted in Aleta Wondo Woreda in Sidama zone, which is located 333 kilometers southeast of Addis Ababa. Administratively, the woreda is subdivided in to 27 rural and 4 urban kebeles (lowest administrative unit). The Woreda, is one of 19 woredas in Sidama zone, and has a total population of 212,459. Among these 184,015 are rural and 28,444 urban dwellers. In the Woreda there are 1 health center, 2 upgrading health centers, 2 medium private clinics and two NGO clinics. The potential health coverage of the Woreda is 29% according to regional health bureau report of 2004/2005.

### Study design

In March 2007 a community based cross sectional study was conducted using both quantitative methods. The study was conducted among all pregnant women who were residing in Aleta Wondo district during the study period. The inclusion criteria were Women, with at least 3 months of current pregnancy, permanent resident of the study area, volunteer to participate and respond to the questionnaire were included. Women who were mentally disabled and severely ill were excluded.

### Sample size and sampling technique

Sample size calculation was made based on the following assumptions pregnant women in the woreda were estimated to be about 4.26% of 212459 = 9050 pregnant women. Proportion of women who know danger signs of pregnancy & childbirth assumed to be 50% because there was no study conducted locally. The margin of error and confidence interval were taken to be 5% and 95% respectively. Based on the above assumption, this gives a sample size of 369. Considering the design effect of 2 and 10% non-response rate, the total sample size became 812.

Multistage sampling procedure was used to select study subjects. First, all the kebeles in the woreda were stratified in to urban and rural. Then 2 urban and 8 rural kebeles were randomly selected for the study. The calculated sample size was proportionally allocated to urban and rural according to their population. Then a census was conducted to register all pregnant women and their gestational age. Based on the above information a sampling list, which enlists all eligible study subjects, was prepared. From the list, pregnant women with gestational age of 3 months and above were included in the survey. As the sample list did not allow simple random sampling, all eligible pregnant women in the selected kebeles were included in the study.

### Measurement

A pre tested Structured interview questionnaire was used for data collection. It was taken from the safe mother hood questionnaire developed by maternal and neonatal health program of JHPIEGO the affiliate of Johns Hopkins University [11], [12], and adapted according to local context and the objectives of the study. Using a pre tested questionnaire the following information were collected. Socio demographic characteristics including: age, marital status, family size, residence (urban versus rural), ethnicity, religion, education, occupation and average monthly family income. The questionnaire included questions gestational age, number of pregnancies, history of still birth and health problems during previous pregnancies. Danger signs during pregnancy, delivery and newborn which require referral and whether the mother follows the following four basic BPACR were asked i) identified a trained birth attendant or ii) health facility for emergency; iii) identified mode of transport for delivery and/or for obstetric emergency; iv) saved money and v) identified blood donor. The women were asked about antenatal care services and number of visits, who attended the ANC, preferred place of delivery.

#### Data collection process

Fifteen community health workers (CHA) who can speak local language were recruited and trained on mapping and conducting households' census with pregnant women conducted a census. Ten-health extension workers from other kebeles interviewed the eligible pregnant women after thorough training on the objective of the study and the questionnaire. Two nurses supervised the data collectors. Data collectors were trained for 4 days by using training manual prepared for this purpose.

#### Data analysis

The collected data were coded, entered, and cleaned, and analyzed by SPSS for windows version 12.0.1. First, simple frequency distribution was calculated. Then those mothers who followed at least two of the five BPACR were considered “well prepared”. The remaining pregnant women were considered “less prepared”. Logistic regression analysis was done to identify factors associated with BPACR.

#### Data quality control

To maintain the validity of the measurement standard questionnaire of safe motherhood was taken and modified based on study interest. The instrument was pre tested on 5% of sample size in Aleta Wondo district that was not included in study and analysis. Modifications were made after pretest. The questions were translated to local language (Sidama language and Amharic) and back translated to English to maintain consistency. The translators were from Woreda education and health department experts on both languages. Training was given to data collectors, supervisors and data entry personnel. Observation and supervision was done throughout the fieldwork, training and data collection process. In addition meeting with each member of the team on a daily basis to discuss performance and give out future work assignments was performed.

Ethical considerations: Ethical approval was obtained from Ethical Review Committee of Jimma University. Letter of support was obtained from the zonal and Woreda health offices before undertaking the study and verbal informed consent was obtained from the respondents before the interview. To document verbal consent, the household ID given during data collection was recorded along with the questionnaire. Due to the large number of individuals that were surveyed and high illiteracy, it was considered impractical to obtain written consent from each study participant, and the institutional review boards approved this procedure. For Privacy and confidentiality, all interviews were conducted in private and all cautions were taken to ensure confidentiality. The right of the respondents to refuse to participate in the study was respected. Respondents were provided information on importance of antenatal care and BPACR.

## Results

### Socio-demographic and obstetric characteristics

Out of 812 pregnant women to be interviewed, 743 were interviewed making a response rate of 92%. The rest were not found for an interview after three repeated visits. The mean±Standard Deviation age of respondents was 25±4 years. Of the respondents 715(96.2%) were currently in marital union and 3.2% were single. Large Majority 644(86.7%) were rural dwellers. By ethnicity 91.8% were Sidamas and 6.1% were Amhara. The major predominant religions include protestant 645(86.8%) and Orthodox Christians 44(5.9%). Educationally 371(49.9%) respondents can't read & write and the rest 50.1% can read and write. Occupationally 710(95.6%) were housewives followed by government employees 12(1.6%). Out of the studied subjects only 568(76.7%) volunteered to tell their income. Regarding pregnancy status majority 62.0% of the respondents had already given birth for two to four children; 20.9% had delivered one child, 17.1% had delivered five and more and the remainders 135(18.2%) were primigravidae. The highest parity was nine. Most (50.6%) of the respondents were in the 3^rd^, 41.5% on 2^nd^, and 7.8% on 1^st^ trimester of pregnancy respectively (Table 1).

**Table 1:** Socio-demographic and obstetric characteristics of pregnant women, Aleta Wondo Woreda, March, 2007.

Characteristics	Number	Percent
Age		
<20	180	24.2
21–25	238	32.0
26–30	271	36.5
>30	54	7.3
Marital status		
Currently in marital union	715	96.2
Currently not in marital union	28	3.8
Residence		
Urban	99	13.3
Rural	644	86.7
Religion		
Protestant	645	86.8
Orthodox	44	5.9
Catholic	29	3.9
Others*	25	3.3
Educational level		
Illiterate	371	49.9
Read and write	105	14.1
Primary (1–4)	96	12.9
5–8	137	18.4
9th and above	34	4.6
Occupation		
House wife	710	95.6
Government employee	12	1.6
House maid	11	1.5
Others**	10	1.3
Ethnicity		
Sidama	682	91.8
Amhara	45	6.1
Others***	16	2.1
Parity		
Para 0	135	18.2
Para 1	127	17.1
Para≥2	481	64.7
Duration of pregnancy		
3–6 months	367	49.4%
7–9 months	376	50.6%
Family size		
≤4	380	51.1
5–6	238	32.0
≥7	125	16.8
Trimester during interview		
First	58	7.8
Second	308	41.5
Third	376	50.7

Other*Muslim, traditional, **occupations like farmer, student, self employee, and jobless, Others***Guraghe,Tigiray, and Silite.

### Antenatal care and preferences during current pregnancy

About 332(44.7%) of respondents attended antenatal care in their current pregnancy. The mean antenatal attendance was 2.4+1.2. Among those pregnant women who attended ANC more than half (61.1%) have had 1–2 visits, 104(31.6%) 3–4 visits and 24(7.3%) more than five visits. The respondents' median gestational age at first antenatal visit was 4.0 month (interquartile range (IQR): 3–5 months).

Majority 228(68.3%) of respondents reported a Health care provider had given health advice during their ANC visit. More than half 184 (55.4%) of respondents were given advice where to go if health problems happen; 138(41.4%) were given where to deliver, 121(36.4%) were advised for arrangement of health care professional to assist in child birth and only 66(20%) were given advice for arrangement for transportation to reach health facility during labor (Table 2).

**Table 2:** Birth preparedness and complication readiness among pregnant women in Aleta Wondo worda March, 2007.

Level of birth preparedness and complication readiness	Number	Percent
Identified a skilled birth attendant	152	20.5
Identified facility for emergency	59	8.1
Arranged transport	57	7.7
Saved money	236	35.5
Identified blood donor	17	2.3
Number of steps taken		
0	353	48.9
1	243	33.7
2	93	12.9
3	22	3.0
4	10	1.4
5	1	0.1
At least 2 steps taken	126	17.0

### Birth preparedness practices

**Figure 1:**
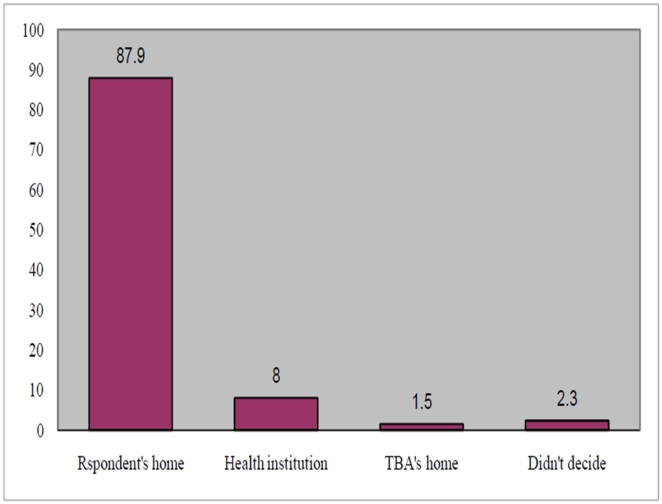
Expected place of delivery among pregnant women in Aleta Wendo Woreda, March 2007.

Only a quarter (20.5%) of pregnant women identified skilled provider. Only 8.1% identified health facility for delivery and/or for obstetric emergencies. Of which 79.7% identified government health facility. Preparedness for transportation was found to be very low (7.7%). Considerable (34.5%) number of families saved money for incurred costs of delivery and emergency if needed (Table 3). Only few (2.3%) identified potential blood donor in case of emergency. Majority of 653(87.9%) of respondents reported that they intended to deliver at home, and only 60(8%) planned to deliver at health facilities. Seventeen (2.3%) of the respondents didn't decide where to give birth (Fig. 1).

**Table 3:** Antenatal care services and preference of birth attendant among pregnant women in Aleta Wondo Woreda, March 20, 2007.

Characteristic	Frequency	Percent
Antenatal care attended during current pregnancy(n = 743)		
Yes	332	44.7
No	411	55.3
Number of ANC visits(n = 329) (Mean±SD; 2.4±1.2)		
1–2	201	61.1
3–4	104	31.6
>5	24	7.3
Personnel checked (n = 329)		
Health professionals	196	59.6
TBA	16	4.9
Community Health Worker	117	35.6
During ANC visit advice given on where to go if health problems happen(n = 331)		
Yes	184	55.6
No	147	44.4
Advise on where to deliver (n = 332)		
Yes	138	41.6
No	194	58.4
Advise on arrangement for transport(n–332)		
Yes	69	20.8
No	263	79.2
Advise on saving money for delivery or emergency (n = 330)		
Yes	66	20.0
No	264	80.0
Advise on arranging blood donor in case of emergency (n = 332)		
Yes	32	9.6
No	300	90.4
Advise on identifying skilled birth attendant (n = 331)		
Yes	121	36.6
No	210	63.4
Expected birth attendant (n = 743)		
Health professional	149	20.1
TBA	232	31.2
Community health worker	101	13.6
Relative/friends	179	24.1
Husband's mother	58	7.8
Don't know	24	3.2

The birth preparedness score was computed from key elements of birth preparedness such as; arrangement for transportation, saving money for delivery, identified skilled attendant to assist at birth, identifying a health facility for emergency and identifying blood donor in case of emergency. Taking at least two steps was considered being well prepared. Accordingly 17% of pregnant women on this study were considered as well prepared for birth and complications.

Compared to the less-prepared pregnant women, the well prepared pregnant women tended to be literate, live in urban area and availed of antenatal services during the current pregnancy. The adjusted multivariate model showed that significant predictors for being well-prepared were maternal availing of antenatal services (OR = 1.91 95% CI; 1.21–3.01) and the current pregnancy was the first for the woman (OR = 6.82, 95% CI; 1.27–36.55) (Table 4).

**Table 4:** Selected characteristics of mothers who were well-prepared* versus those who were less-prepared (n = 743).

Characteristics	Number (Percent)		Unadjusted OR	Adjusted OR^†^
Family size	Less prepared (n = 616)	Well prepared (n = 126)	OR(95% CI)	OR(95%CI)
= 4	306(49.7)	73(57.9)	0.64(0.35–1.16)	0.58(0.31–1.10)
5–6	208(33.8)	30(23.8)	1.06(0.63–1.78)	1.06(0.56–2.01)
= 7	102(16.6)	23(18.3)	1.0	1.0
Marital status				
In marital union	593(96.3)	121(96.0)	0.94(0.35–2.52)	1.27(0.33–4.88)
Not in marital union	23(3.7)	5(4.0)	1.0	1.0
Residence				
Rural	545(88.5)	98(77.8)	0.46(0.28–0.74)	0.53 (0.27–1.04)
Urban	71(11.5)	28(22.2)	1.0	
First pregnancy				
Yes	105(17.0)	29(23.0)	1.45(0.91–2.32)	6.82(1.27–36.55)^$^
No	511(83.0)	97(77.0)	1.0	
History of still birth				
Yes	35(6.8)	7(7.0)	1.02(0.44–2.37)	1.14(0.48–2.74)
No	476(93.2)	93(93.0)	1.0	
Education				
Illiterate	323(52.4)	47(37.3)	0.54(0.36–0.80)	0.64(0.40–1.01)
Literate	293(47.6)	79(62.7)	1.0	
Occupation				
Employed	16(2.6)	7(5.6)	2.21(0.89–5.48)	1.69(0.54–5.24)
Unemployed	600(97.4)	119(94.4)	1.0	
Aware of at least two danger-sign during pregnancy				
Yes	439(71.4)	76(60.3)	0.61(0.41–0.91)	0.63(0.37–1.08)
No	176(28.6)	50(39.7)	1.0	
Aware of at least two danger-sign during delivery				
Yes	257(42.0)	47(37.3)	0.82(0.55–1.22)	1.09(0.65–1.85)
No	355(58.0)	79(62.7)	1.0	
Aware of at least two danger-sign after delivery				
Yes	389(63.4)	72(57.1)	0.77(0.52–1.14)	0.98(0.56–1.69)
No	225(36.6)	54(42.9)	1.0	
Attended ANC during current pregnancy				
Yes	258(42.0)	73(57.9)	1.91(1.29–2.81)	1.91(1.21–3.01)**
No	357(58.0)	53(42.1)	1.0	
Age				
= 25	342(55.5)	75(59.5)	1.18(0.80–1.74)	0.86(0.52–1.42)
>25	274(44.5)	51940.5)	1.0	

CI = Confidence Interval, OR = Odds ratio; ANC = Antenatal care, *Any 2 of 5 steps: identified a trained birth attendant, identified a health facility, arranged for transport, identified blood donor and saved money for emergency; ^$^ Significant at p<0.05, **significant at p<0.01, ^†^Adjusted for all the independent variables indicated in the table.

## Discussion

In this study several important findings were observed. Less than a quarter of pregnant women were well prepared for delivery and emergency obstetric care; availing antenatal care and being pregnant for the first time were predictors of birth preparedness and complication readiness. The finding of this study is consistent with previous study [26] and reinforce efforts to increase BPACR should focus on availing antenatal care services.

Studies have indicated the relation between BPACR and skilled birth attendant. The findings showed that BPACR increases skilled birth attendants [15], [26]. In this study however only 17% of the pregnant women were well prepared, implying the importance of interventions to increase BPACR in the setup. Similar to other study conducted in India [26] in this study those women who attended antenatal care service were well prepared than those who did not attend. This signifies that antenatal care services visits are an opportunity to inform pregnant women and help to plan for the important components of BPACR. Our finding indicated that women were not informed well all the components of BPACR utterly. This implies the importance of training for health providers on how to advise pregnant women on components of BPACR.

In our study women with first pregnancy were more prepared than their counterparts. This could be due to high risk perception of such women than those who had experience. This shows that increasing risk perception might help in improving BPACR.

In our study only few (7.7%) pregnant women made adequate arrangements for transportation to a health facility in case of an obstetric emergency. This is far less than findings from Burkina Faso's (35%) [15] and India (29.5%) [26].This could be differences in the local contexts. In our setup the community uses traditional ways such as donkey cart and local stretchers to carry patients to facilities. Unavailability of roads in some of the rural setups plays role. Therefore messages on BPACR should be tailor to the local contexts and doe able messages.

In this study a large proportion 87.9% of women reported that they intended to give birth in their home, and only 8% planned to deliver in health facilities. Furthermore only 20.1% of pregnant women in this study planned to deliver by assistance of skilled provider. While increasing knowledge to prepare for birth and emergencies is important, efforts are required to identify barriers for use of health facilities and skilled attendants at birth. Since most of pregnant women relay on TBAs it is important to make TBAs allies of the health system. They can be used as referral linkage by escorting pregnant women to health facilities.

The strength of the study includes it is a community based; census was conducted before data collection to identify currently pregnant women and also large sample size was used. The limitations of the study are: since the participants have not completed their pregnancies, they may not yet have had the opportunity or need to make arrangements related to BPACR. Pregnant women may not able to report whether they used services that they have not yet needed. There could be social desirability bias; to reduce this we used health extension workers from other areas. Finally previous study [16] has indicated that pregnant women were more likely to report planning to give birth with the assistance of a skilled provider, which might not reflect the true result.

This study revealed that only few of pregnant women were well prepared for delivery and obstetric complication, a large majority of pregnant women planned to deliver at home where the presence of skilled attendants is uncertain. Few pregnant women planned to have assisted by skilled attendant. In addition only small percentage of pregnant women arranged financial preparations and identified transportation to reach health facility to deliver. The present study shows availing antenatal care services and being pregnant for the first time affected BPACR.

Therefore the MOH, regional health bureau, zonal health department, Woreda health office as well as other partner organizations that are working in areas of maternal health should come up with strategies to improve birth preparedness at individual and community level. Effort to increase BPACR should focus on availing antenatal care.
